# Mechanical Fatigue Performance of Patient-Specific Polymer Plates in Oncologic Mandible Reconstruction

**DOI:** 10.3390/jcm11123308

**Published:** 2022-06-09

**Authors:** Julian Lommen, Lara Schorn, Christoph Sproll, Norbert R. Kübler, Luis Fernando Nicolini, Ricarda Merfort, Ayimire Dilimulati, Frank Hildebrand, Majeed Rana, Johannes Greven

**Affiliations:** 1 Department of Oral-, Maxillofacial and Facial Plastic Surgery, University Hospital Düsseldorf, Moorenstr. 5, 40225 Dusseldorf, Germany; julian.lommen@med.uni-duesseldorf.de (J.L.); christoph.sproll@med.uni-duesseldorf.de (C.S.); kuebler@med.uni-duesseldorf.de (N.R.K.); rana@med.uni-duesseldorf.de (M.R.); 2 Department of Orthopedics, Trauma and Reconstructive Surgery, RWTH Aachen University Hospital, Pauwelsstreet 30, 52074 Aachen, Germany; lnicolini@ukaachen.de (L.F.N.); rmerfort@ukaachen.de (R.M.); ayimire.dilimulati@alumni.fh-aachen.de (A.D.); fhildebrand@ukaachen.de (F.H.); jgreven@ukaachen.de (J.G.)

**Keywords:** reconstruction plate, segmental mandibulectomy, polymer plate, mechanical properties, polyetheretherketone, polyetherketoneketone, polyphenylsulfone, computer-aided design (CAD), computer-aided manufacturing (CAM)

## Abstract

Mandible defects are conventionally reconstructed using titanium plates. However, titanium causes metallic artifacts which impair radiological imaging. This study aims at evaluating mechanical fatigue of radiolucent fiber-reinforced polyetheretherketone (f-PEEK), polyetheretherketone (PEEK), polyetherketoneketone (PEKK), and polyphenylsulfone (PPSU) polymer plates for mandible reconstruction. A total of 30 plates (titanium [n = 6], f-PEEK [n = 6], PEEK [n = 6], PEKK [n = 6], PPSU [n = 6]) were implanted in synthetic mandibulectomized polyurethane mandibles. Servo-pneumatic mechanical testing with cyclic application of 30–300 N at 3 Hz was conducted. Bite forces were 70% on the unresected and 30% on the resected side. Total number of cycles was set to 250,000. Testing was aborted in case of plate or screw failure. Axial load to failure was tested with a speed of 1 mm/s. Kruskal–Wallis and Dunn’s post hoc tests were used. Titanium, f-PEEK, and PEEK showed no failure in fatigue testing and PPSU (*p* < 0.001) failed against titanium, f-PEEK, PEEK, and PEKK. Titanium allowed the highest load to failure compared to f-PEEK (*p* = 0.049), PEEK (*p* = 0.008), PEKK (*p* < 0.001), and PPSU (*p* = 0.007). f-PEEK, PEEK, and PEKK withstood expected physiological bite force. Although titanium plates provided the highest fatigue strength, f-PEEK and PEEK plates showed no failure over 250,000 chewing cycles indicating sufficient mechanical strength for mandible reconstruction.

## 1. Introduction

Surgical treatment of patients with oral squamous cell carcinoma (OSCC) often requires substantial osseous resection including segmental mandibulectomy when medullary bone infiltration is diagnosed [1]. In those cases, surgical reconstruction of the mandible is commonly conducted by implantation of rigid titanium reconstruction plates to restore the mandibular continuity. These plates can be conventionally bent intraoperatively or patient-specifically and offer a high degree of mechanical stability [2]. Computer-aided designing of patient-specific titanium implants (PSI) using precise selective laser melting techniques improves postoperative masticatory function, esthetics, and quality of life in patients [3]. Due to sophisticated fitting accuracy, the use of PSI in mandibular reconstruction is widely accepted [4]. However, titanium plates cause significant streak and blooming artifacts in computer tomography (CT) images thwarting thorough radiological assessment of implant surrounding tissues [5]. This phenomenon disadvantageously affects postoperative radiological tumor follow-up as well as adjuvant radiotherapy strongly dependent on a high-resolution, artifact-free planning CT [6]. Furthermore, titanium implants cause a radiation dose enhancement affecting the immediate surrounding tissues [7].

As a counterpart to this, it could be shown that various types of polymer reconstruction plates consisting of polyetheretherketone (PEEK), polyetherketoneketone (PEKK), polyphenylsulfone (PPSU), and polyethylene (PE) cause significantly fewer artifacts in CT images leading to better image quality [5]. Because bite forces after segmental mandibulectomy remain limited, presumably frangible polymer reconstruction materials have become of enormous scientific interest [8]. With this background, even glass fiber-reinforced composite plates which have been shown to provide less fatigue strength and stiffness compared to titanium plates might be an option for mandibular reconstructions [9]. The polyaromatic semi-crystalline PEEK is a tough, rigid, and biocompatible osteosynthesis material widely used in cranioplasty and facial reconstructive surgery [10,11]. PEKK is an elastic polymer with good shock absorbance properties and mechanical strength, whereas PPSU is a heat-resistant and stable polymer [12,13]. There are no scientific data on the biomechanical strength of PEEK, carbon fiber-reinforced polyetheretherketone (f-PEEK), PEKK, and PPSU used as reconstruction plates after segmental mandibulectomy.

Consequently, the aim of the present study was to evaluate mechanical fatigue of patient-specific PEEK, f-PEEK, PEKK, and PPSU reconstruction plates compared to titanium plates. We hypothesize that polymer plates endure a comparable number of cycles until failure providing sufficient stability to withstand expected physiological bite forces after segmental mandibulectomy.

## 2. Materials and Methods

### 2.1. Test Specimens

A total number of 30 synthetic polyurethane mandibles (SYNBONE^®^, Zizers, Switzerland) with the model number 8950 were used in this study. As described by numerous other studies these mandibles have a striking resemblance to human bone and are as similar as possible to the original anatomical shape [9,14,15,16]. All mandibles used in this study were standardized models from the same lot number. None of the mandibles had missing teeth prior to segmental mandibulectomy.

### 2.2. Plates and Screws

**Figure 1:**
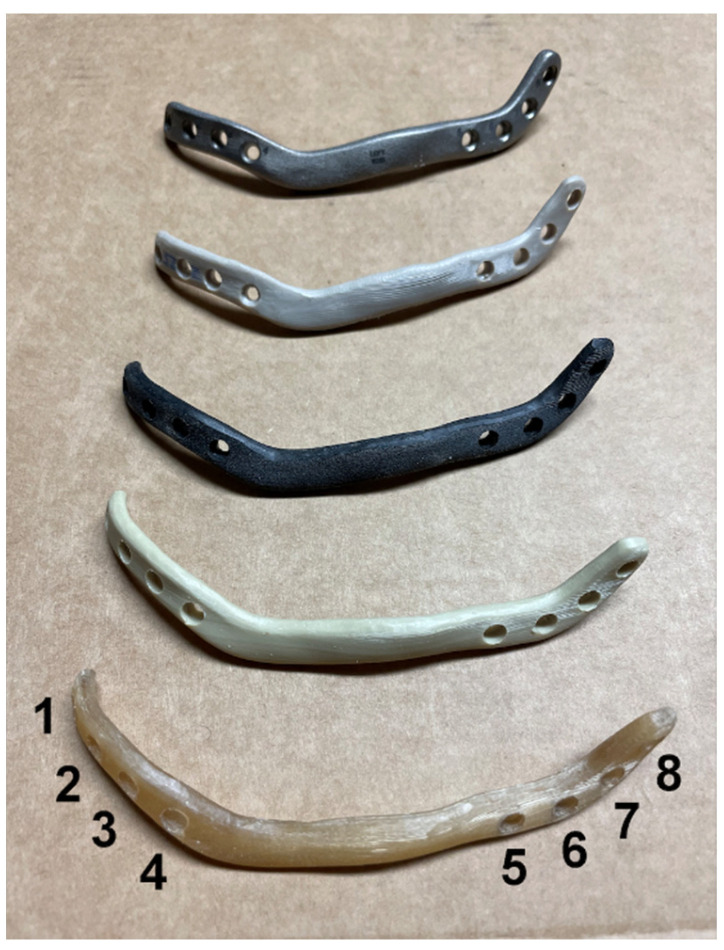
Display of the five different plates used for defect bridging. All plates in this trial are identical in design and dimensions. From top: titanium, polyetheretherketone (PEEK), fiber-reinforced polyetheretherketone (f-PEEK), polyetherketoneketone (PEKK), and polyphenylsulfone (PPSU). The numbers 1–8 symbolize the different drilling holes for implant orientation and selection of the required screw length.

Titanium (Ti6Al4V ELI (Grade 23), SLM Solutions, Lübeck, Germany), PEEK (VESTAKEEP^®^ i4 3DF-T, Evonik, Essen, Germany), f-PEEK (TECAFIL PEEK MT CF 30, Ensinger, Seewalchen, Austria), PEKK (PEKK Filament, Kumovis, Munich, Germany), and PPSU (Veriva^®^ PPSU, Solvay, Hanover, Germany) reconstruction plates were used in this trial (Figure 1). All plates are identical in design and dimensions and only differ in the used material. For rigid fixation, all plates contained four screw holes on both sides of the defect marked as positions 1–8 (Figure 1). Plate thickness was 3.0 mm for all plates. Bicortical self-retaining titanium screws with a diameter of 2.7 mm and a 2.2 × 105 mm drill without stop were used for plate fixation. For respective screw parameters see Table 1.

### 2.3. Virtual Planning, Shaping, and Manufacturing of Reconstruction Plates

**Figure 2:**
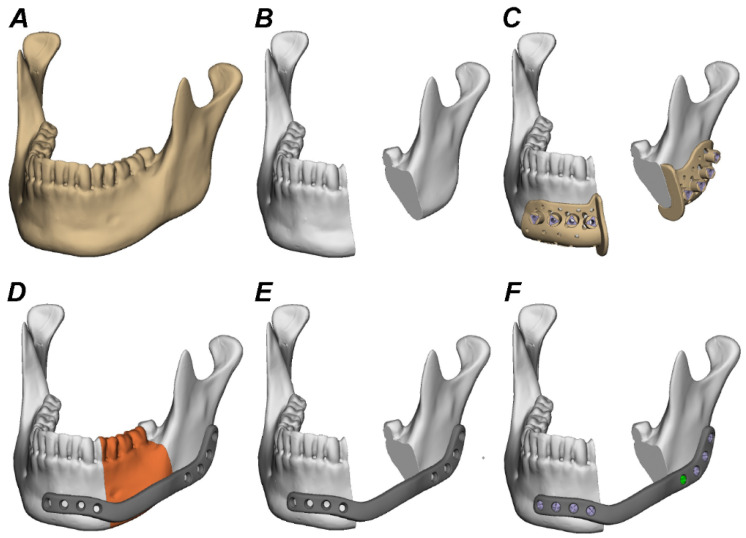
Segmentation, 3D reconstruction, and virtual planning of the mandibular defect size and plate design. (**A**–**C**), Digital image of the scanned polyurethane mandible before and after defining the defect size and planning the positioning of the resection guides. (**D**–**F**), Assessment of the adequate dimensions for patient-specific titanium and polymer plates using the medical modeling software (Geomagic© Freeform Plus© from 3D Systems ©, Rock Hill, SC, USA).

ATOS Core 135 MV135 scanner (GOM GmbH, Braunschweig, Germany) was used for computer-aided design and manufacturing (CAD/CAM) of titanium, PEEK, f-PEEK, PEKK, and PPSU plates. Segmentation, 3D reconstruction, and virtual planning was conducted using the software Individual Patient Solution, IPS Gate^®^ (KLS Martin Group^®^, Tuttlingen, Germany) (Figure 2). The 3D virtual model was subsequently converted to stereolithography (STL) image files using Mimics 21.0© (Materialise NV, Belgium). Webinar-based (Microsoft© Teams, Redmond, WA, USA) virtual surgery between surgeons and engineers from KLS Martin© defined defect size of segmental mandibulectomy (Figure 2). Dimensions of titanium and polymer plates were defined using Geomagic© Freeform Plus© (3D Systems©, Rock Hill, SC, USA) (Figure 2). Titanium plates were manufactured using an additive selective laser melting (SLM) procedure. f-PEEK, PEEK, PEKK, and PPSU plates were manufactured using additive fused filament fabrication (FFF). Resection guides were laser-sintered from polyamide (PA 2200).

### 2.4. Plate Fixation to Test Specimens

**Figure 3:**
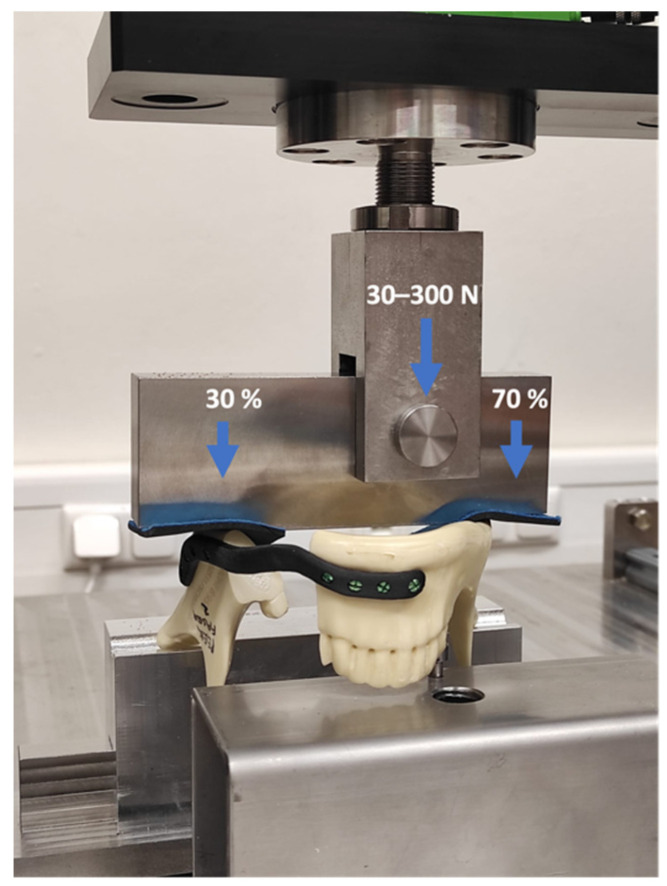
Display of servo-hydraulic mechanical testing with a MTS Bionix (Eden Prairie, MN, USA) testing machine. Cyclical loading of bite forces between 30 and 300 N with distribution of 30% of maximum force on the resected and 70% on the unresected side of the mandible.

For segmental mandibulectomy individualized polyamide resection-guides and rotating burrs were utilized. The first and second molar as well as premolar teeth were resected. No plate prepositioning before resection was conducted. For plate fixation, bicortical self-retaining titanium screws specifically positioned according to the plate design were used (Figure 3).

### 2.5. Mechanical Testing

Based on prior studies simulating bite forces after segmental mandibulectomy controlled application of 300 N in the premolar region of the mandibles was conducted [17]. As current literature suggests bite forces were set to 70% on the unresected and 30% on the resected side of the specimen using a see-saw device [16]. Fatigue mechanical testing was carried out with a custom-made servo pneumatic test stand (DynaMess, Stolberg, Germany) with cyclical application of bite forces of up to 300 N. The force applied on the specimen was controlled and recorded by a force transducer that provides a maximum error of 1% relative to the target value. Fixation of mandibles in the testing machine is displayed in Figure 3. The minimum preload force of 30 N was set for practical reasons to prevent dislocation of the mandibles. Frequency was adjusted to 3 Hz. The total number of cycles was set to 250,000 (and 500,000 for titanium) which roughly represents the amount of chewing cycles per year [16] and was recorded at a frequency of 100 Hz [18]. Testing was stopped in case of plate or screw failure or significant deformation of osteosynthesis materials; cycles were then recorded up to the point of failure. If 250,000 cycles of fatigue loading were completed without failure, the bone–implant construct was exposed to axial load to failure compression testing with a testing speed of 1 mm/s [19].

### 2.6. Statistical Analysis

Statistical analyses were conducted using Microsoft Excel^©^ (Microsoft Corporation, Redmond, WA, USA) and IBM^©^ SPSS^©^ Statistics (Version 22, IBM GmbH, Ehningen, Germany). Levene’s test was used to assess homogeneity of variance. Kruskal Wallis test and Wilcoxon signed-rank test were used for comparative analyses between groups. Post-hoc Bonferroni analysis was done using Dunn’s test. A *p*-value < 0.05 was considered statistically significant.

## 3. Results

### 3.1. Fatigue Test

**Figure 4:**
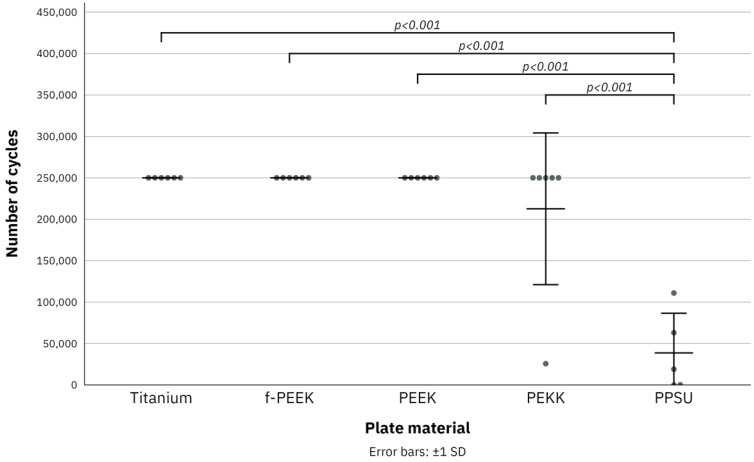
Boxplot diagram of the results of fatigue test for titanium, fiber-reinforced polyetheretherketone (f-PEEK), polyetheretherketone (PEEK), polyetherketoneketone (PEKK) and polyphenylsulfone (PPSU) plates over the course of 250,000 cycles. Error bars: ±1 standard deviation. Jitter display for numbers of the same value.

In total 29 plates were tested in the fatigue test over the course of 250,000 cycles to analyze plate failure. All titanium, f-PEEK, and PEEK plates (n = 6) showed no signs of failure after completion of 250,000 cycles. There was no statistical difference between these three plate materials. Of all tested PEKK plates (n = 6), five showed no signs of failure and one plate broke after 25,701 cycles. All PPSU plates (n = 5) broke during testing after as little as 6 cycles and lasting a maximum of 110,997 cycles before failure. Since all five PPSU plates broke quite early, the last PPSU plate was spared for use in load to failure testing. Statistical differences were found between PPSU and titanium (*p* < 0.001), f-PEEK (*p* < 0.001), PEEK (*p* < 0.001), and PEKK (*p* < 0.001) plates. A summary of the results of the fatigue test is given in Table 2 and Figure 4.

### 3.2. Load to Failure Test

**Figure 5:**
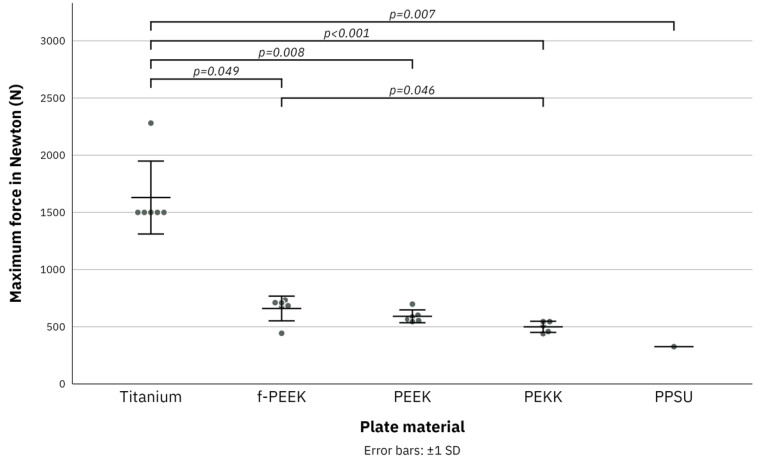
Boxplot diagram of the results of load to failure test for titanium, fiber-reinforced polyetheretherketone (f-PEEK), polyetheretherketone (PEEK), polyetherketoneketone (PEKK), and polyphenylsulfone (PPSU) plates for maximum force of 2500 Newton [N]. Error bars: ±1 standard deviation. Jitter display for numbers of the same value.

None of the six titanium plates broke in a load to failure test to a maximum force of 1500 N. One titanium plate was tested at a maximum force of 2281 N with no signs of failure of the plate but failure of the specimen. f-PEEK plates broke at a maximum force of 443 N and PEEK plates broke at a maximum force of 545 N. PEKK plates broke at a maximum force of 440 N with one plate already breaking over the course of the fatigue test. Since five PPSU plates already broke in the fatigue test only one PPSU plate could be tested in the load to failure test. PPSU broke at a maximum force of 326 N. Statistical differences were found between PPSU and titanium (*p* = 0.007), PEKK and titanium (*p* < 0.001), PEKK and f-PEEK (0.046), PEEK and titanium (*p* = 0.008), and f-PEEK and titanium (*p* = 0.049). A summary of the results of the load to failure test is given in Table 3 and Figure 5.

## 4. Discussion

The use of durable patient-specific titanium plates for reconstruction of the mandible in patients with OSCC is common practice. A recent study found that CAD/CAM titanium reconstruction plates provide higher fatigue strength compared to miniplates and higher stiffness compared to manually bend reconstruction plates [14]. Despite their conventional use in oncological head and neck reconstruction, metallic artifact formation in CT and MRI scans as well as impediments in radiation dose calculations in adjuvant radiation therapy remain a serious medical problem [20,21]. In the wake of scientific efforts to counter these problems, polymer materials are increasingly being investigated as possible substitutes. Just recently it was shown that the polymer materials PEEK, PEKK, PPSU, and PE cause significantly fewer artifacts in CT imaging when used as mandible reconstruction plates compared to titanium tested on human cadavers [5].

However, this alone does not justify their application as interchangeable plates in reconstructive surgery as only few studies have evaluated the biomechanical properties of such polymers. A recent study found that despite glass fiber-reinforced composite (GFRC) reconstruction plates providing less stiffness compared to CAD/CAM titanium plates, primary stability for mandible reconstruction with an osseous free flap is sufficient [9]. The results of the present study show that carbon fiber reinforced PEEK plates provide comparable fatigue strength under cyclic chewing forces. This is a very promising result since the average time for adequate bone union of a vascularized osseous fibula flap was found to be 21.3 weeks in another recent study [22]. Under this scientific assumption, polymer plates in mandible reconstruction would only need to provide primary stability for approximately 6 months until sturdy bone union is achieved. As the study by Schupp et al., (2007) suggests, the number of annual chewing cycles with considerable bite forces is estimated to be 250,000 [16,23]. Therefore, the results of the present study indicate that f-PEEK and PEEK reconstruction plates offer sufficient fatigue strength and stiffness to endure regular bite forces at least over the course of one year. Since maximum bite forces of adult men and women are reported to be 284.90 N and 304.96 N respectively, the presented data show that not only f-PEEK and PEEK but also PEKK plates withstood simulated physiological bite forces [24]. Reduced bites forces after segmental mandibulectomy and the small proportion of patients being fully prosthodontically rehabilitated in the long-term warrant consideration of polymer plates as possible future alternatives for titanium plates [1]. However, the data of the presented study only evaluated fatigue strength of polymer plates in one defect location of the body of the mandible. In the future, it is advisable to also test fatigue strength of polymer plates bridging larger mandibular defects, especially when the midline is crossed. Since time of postoperative radiation therapy in tumor patients at advanced clinical stage should ideally not exceed 6 weeks, mechanical properties of f-PEEK and PEEK plates presumably suffice [25]. In case of a two-stage surgical approach, prolonged plate stability becomes particularly important to prevent plate fracture before reconstruction with a bone graft. When plates are combined with an osseous free flap, a sufficient time frame for primary stability and bone union also appears likely. Some studies suggest stiffness being the most important aspect of stability of osteosynthesis materials [26]. All screws and plates in the presented study were of locking-type which further increases mechanical stiffness. None of the inserted screws in this study broke. However, interaction between polymer locking plates and titanium locking screws needs to be regarded as weaker compared to fully-titanium components. There are studies indicating that overly stiff CAD/CAM titanium plates could obstruct bone union by preventing a certain amount of bone fragment movement which is considered beneficial [14,27]. Therefore, hypothetically, the weaker polymer plates may support ossification of bone grafts to a certain extent. However, it needs to be clarified that the present study did not evaluate bone union or interfragmentary movement—a strong predictor of pseudarthrosis—of a bone graft fixated with polymer plates. Insertion of a bone graft after segmental mandibulectomy may stabilize the anterior and posterior bony ends and possibly deceases torque of the reconstruction plate [28]. This might further increase fatigue strength of polymer plates and should be tested in future trials.

At this time, it remains scientifically unclear which factors positively influence bone healing after segmental mandibulectomy. Since all titanium and polymer plates in the present study were manufactured with identical dimensions and inserted with the same type of screw, system comparability of results is high. However, the described individual manufacturing of patient-specific polymer plates poses the disadvantage that plates cannot be adjusted during surgery. Therefore, precise preoperative planning is a key factor for an excellent outcome. It has been indicated that plate thickness might be a relevant factor for soft tissue complications such as plate exposure [29]. The study by Rendenbach et al., (2019) used 2-mm thick GFRC plates in their tests [9]. This is slightly thinner than the 3-mm polymer plates used in the present study. Since conventional CAD/CAM titanium plates typically show a thickness between 2 and 3 mm, it seems unlikely that the polymer materials will cause a drastic increase in soft tissue complications even though this has not yet been investigated. The presented data have their limitations as they have not been tested under in vivo conditions. Patient gender, age, comorbidities as well as soft tissue management have not been evaluated in this study. Moreover, the number of residual teeth in patients with OSCC prior to surgery varies. As the dentition is a strong predictor for expected residual bite forces after segmental mandibulectomy, it is likely that numerous patients present with lower bite forces compared to the forces tested in this study [30]. Consequently, polymer plate fracture especially in combination with a bone graft might be less likely in vivo.

In considering the immediate scientific future, recent studies have focused on tissue engineering procedures based on the release of growth factors from polymer materials to improve bone healing [31,32]. The use of bone morphogenetic protein-2 (rhBMP-2) with a collagen carrier was shown to provide good osseous regeneration even without concomitant bone materials [33]. The study group of the present trial previously showed that insoluble bovine collagenous bone matrices have a better release kinetic of the model protein fluorescein conjugated bovine serum albumin (FITC-BSA) when coated with medium molecular size polymers [34]. Such coatings might also be possible for polymer reconstruction plates of the mandible shown in the present study. It remains to be seen how such polymer plates perform in vivo.

## 5. Conclusions

CAD/CAM titanium reconstruction plates still provide a higher fatigue strength compared to f-PEEK, PEEK, PEKK, and PPSU polymers. However, f-PEEK and PEEK plates showed no failure over the simulated 250,000 chewing cycles certifying sufficient maximal mechanical strength and durability under cyclic physiological load for mandible reconstruction. Future trials need to assess interaction between the polymers and soft tissues on a molecular level.

## Figures and Tables

**Table 1:** List of all screw types, positions and respective lengths.

Screw Type	Position	Screw Length (mm)
Ø 2.7 mm, locking (bicortical)	1	17
Ø 2.7 mm, locking (bicortical)	2	17
Ø 2.7 mm, locking (bicortical)	3	15
Ø 2.7 mm, locking (bicortical)	4	17
Ø 2.7 mm, locking (bicortical)	5	13
Ø 2.7 mm, locking (bicortical)	6	11
Ø 2.7 mm, locking (bicortical)	7	11
Ø 2.7 mm, locking (bicortical)	8	9

**Table 2:** Results of the fatigue test. N/A: Plate used in load to failure test.

Plate Number	Plate Material	Number of Cycles	Plate Failure	Screw Failure
1	Titanium	250,000	No	No
2	Titanium	250,000	No	No
3	Titanium	250,000	No	No
4	Titanium	250,000	No	No
5	Titanium	250,000	No	No
6	Titanium	250,000	No	No
1	f-PEEK	250,000	No	No
2	f-PEEK	250,000	No	No
3	f-PEEK	250,000	No	No
4	f-PEEK	250,000	No	No
5	f-PEEK	250,000	No	No
6	f-PEEK	250,000	No	No
1	PEEK	250,000	No	No
2	PEEK	250,000	No	No
3	PEEK	250,000	No	No
4	PEEK	250,000	No	No
5	PEEK	250,000	No	No
6	PEEK	250,000	No	No
1	PEKK	250,000	No	No
2	PEKK	25,701	Yes	No
3	PEKK	250,000	No	No
4	PEKK	250,000	No	No
5	PEKK	250,000	No	No
6	PEKK	250,000	No	No
1	PPSU	285	Yes	No
2	PPSU	6	Yes	No
3	PPSU	19,011	Yes	No
4	PPSU	63,075	Yes	No
5	PPSU	110,997	Yes	No
6	PPSU	N/A	N/A	N/A

**Table 3:** Results of the load to failure test. N/A: Plate broke in fatigue test.

Plate Number	Plate Material	Maximum Force (N)
1	Titanium *	2281
2	Titanium **	1500
3	Titanium **	1500
4	Titanium **	1500
5	Titanium **	1500
6	Titanium **	1500
1	f-PEEK	443
2	f-PEEK	711
3	f-PEEK	733
4	f-PEEK	679
5	f-PEEK	684
6	f-PEEK	709
1	PEEK	698
2	PEEK	565
3	PEEK	586
4	PEEK	601
5	PEEK	554
6	PEEK	545
1	PEKK	440
2	PEKK	N/A
3	PEKK	509
4	PEKK	545
5	PEKK	545
6	PEKK	458
1	PPSU	N/A
2	PPSU	N/A
3	PPSU	N/A
4	PPSU	N/A
5	PPSU	N/A
6	PPSU	326

* Failure of the polyurethan mandible. ** Maximum force was set to 1500 N, no failure occurred.

## Data Availability

Not applicable.

## References

[1] Sproll C.K., Holtmann H., Schorn L.K., Jansen T.M., Reifenberger J., Boeck I., Rana M., Kübler N.R., Lommen J. (2020). Mandible handling in the surgical treatment of oral squamous cell carcinoma: Lessons from clinical results after marginal and segmental mandibulectomy. Oral Surg. Oral Med. Oral Pathol. Oral Radiol..

[2] Möllmann H.L., Apeltrath L., Karnatz N., Wilkat M., Riedel E., Singh D.D., Rana M. (2021). Comparison of the Accuracy and Clinical Parameters of Patient-Specific and Conventionally Bended Plates for Mandibular Reconstruction. Front. Oncol..

[3] Vignesh U., Mehrotra D., Howlader D., Singh P.K., Gupta S. (2019). Patient Specific Three-Dimensional Implant for Reconstruction of Complex Mandibular Defect. J. Craniofacial Surg..

[4] Toto J.M., Chang E.I., Agag R., Devarajan K., Patel S.A., Topham N.S. (2015). Improved operative efficiency of free fibula flap mandible reconstruction with patient-specific, computer-guided preoperative planning. Head Neck.

[5] Lommen J., Schorn L., Sproll C., Haussmann J., Kübler N.R., Budach W., Rana M., Tamaskovics B. (2022). Reduction of CT Artifacts Using Polyetheretherketone (PEEK), Polyetherketoneketone (PEKK), Polyphenylsulfone (PPSU), and Polyethylene (PE) Reconstruction Plates in Oral Oncology. J. Oral Maxillofac. Surg..

[6] Bojechko C., Hua P., Sumner W., Guram K., Atwood T., Sharabi A. (2021). Adaptive replanning using cone beam CT for deformation of original CT simulation. J. Med. Radiat. Sci..

[7] Friedrich R.E., Todorovic M., Heiland M., Scheuer H.A., Krüll A. (2012). Scattering effects of irradiation on surroundings calculated for a small dental implant. Anticancer Res..

[8] Maurer P., Pistner H., Schubert J. (2006). Computer assisted chewing power in patients with segmental resection of the mandible. Mund Kiefer Gesichtschir..

[9] Rendenbach C., Steffen C., Sellenschloh K., Heyland M., Morlock M.M., Toivonen J., Moritz N., Smeets R., Heiland M., Vallittu P.K. (2019). Patient specific glass fiber reinforced composite versus titanium plate: A comparative biomechanical analysis under cyclic dynamic loading. J. Mech. Behav. Biomed. Mater..

[10] Zhang J., Tian W., Chen J., Yu J., Zhang J., Chen J. (2019). The application of polyetheretherketone (PEEK) implants in cranioplasty. Brain Res. Bull..

[11] Panayotov I.V., Orti V., Cuisinier F., Yachouh J. (2016). Polyetheretherketone (PEEK) for medical applications. J. Mater. Sci. Mater. Med..

[12] Alqurashi H., Khurshid Z., Syed A.U.Y., Rashid Habib S., Rokaya D., Zafar M.S. (2021). Polyetherketoneketone (PEKK): An emerging biomaterial for oral implants and dental prostheses. J. Adv. Res..

[13] Díez-Pascual A.M., Díez-Vicente A.L. (2014). Effect of TiO(2) nanoparticles on the performance of polyphenylsulfone biomaterial for orthopaedic implants. J. Mater. Chem. B.

[14] Rendenbach C., Sellenschloh K., Gerbig L., Morlock M.M., Beck-Broichsitter B., Smeets R., Heiland M., Huber G., Hanken H. (2017). CAD-CAM plates versus conventional fixation plates for primary mandibular reconstruction: A biomechanical in vitro analysis. J. Craniomaxillofac. Surg..

[15] Steiner T., Raith S., Scherer E., Mücke T., Torsiglieri T., Rohleder N.H., Eder M., Grohmann I., Kesting M., Bier H. (2015). Which kind of frontal mandibulotomy is the smartest? A biomechanical study. J. Craniomaxillofac. Surg..

[16] Schupp W., Arzdorf M., Linke B., Gutwald R. (2007). Biomechanical testing of different osteosynthesis systems for segmental resection of the mandible. J. Oral Maxillofac. Surg..

[17] Slagter A.P., Bosman F., van der Glas H.W., van der Bilt A. (1993). Human jaw-elevator muscle activity and food comminution in the dentate and edentulous state. Arch. Oral Biol..

[18] Nicolini L.F., Kobbe P., Seggewiß J., Greven J., Ribeiro M., Beckmann A., Da Paz S., Eschweiler J., Prescher A., Markert B. (2022). Motion preservation surgery for scoliosis with a vertebral body tethering system: A biomechanical study. Eur. Spine J..

[19] Brandes L.L., Nicolini L.F., Greven J., Lichte P., Stopinski T.T., Sattler M., Hildebrand F., Pishnamaz M. (2021). Biomechanical Performance of BoneHelix(^®^) Compared with Elastic Stable Intramedullary Nailing (ESIN) in a Pediatric Tibia Fracture Model. Life.

[20] Radzi S., Cowin G., Robinson M., Pratap J., Volp A., Schuetz M.A., Schmutz B. (2014). Metal artifacts from titanium and steel screws in CT, 1.5T and 3T MR images of the tibial Pilon: A quantitative assessment in 3D. Quant. Imaging Med. Surg..

[21] Kovacs D.G., Rechner L.A., Appelt A.L., Berthelsen A.K., Costa J.C., Friborg J., Persson G.F., Bangsgaard J.P., Specht L., Aznar M.C. (2018). Metal artefact reduction for accurate tumour delineation in radiotherapy. Radiother. Oncol..

[22] Liu S., Tao S., Tan J., Hu X., Liu H., Li Z. (2018). Long-term follow-up of fibular graft for the reconstruction of bone defects. Medicine.

[23] Bates J.F., Stafford G.D., Harrison A. (1975). Masticatory function-a review of the literature: (II) Speed of movement of the mandible, rate of chewing and forces developed in chewing. J. Oral Rehabil..

[24] Takaki P., Vieira M., Bommarito S. (2014). Maximum bite force analysis in different age groups. Int. Arch. Otorhinolaryngol..

[25] Franco R., de Matos L.L., Kulcsar M.A.V., de Castro-Júnior G., Marta G.N. (2020). Influence of time between surgery and postoperative radiation therapy and total treatment time in locoregional control of patients with head and neck cancer: A single center experience. Clinics.

[26] Henschel J., Tsai S., Fitzpatrick D.C., Marsh J.L., Madey S.M., Bottlang M. (2017). Comparison of 4 Methods for Dynamization of Locking Plates: Differences in the Amount and Type of Fracture Motion. J. Orthop. Trauma.

[27] Trainotti S., Raith S., Kesting M., Eichhorn S., Bauer F., Kolk A., Lethaus B., Hölzle F., Steiner T. (2014). Locking versus nonlocking plates in mandibular reconstruction with fibular graft--a biomechanical ex vivo study. Clin. Oral Investig..

[28] Park S.M., Lee J.W., Noh G. (2018). Which plate results in better stability after segmental mandibular resection and fibula free flap reconstruction? Biomechanical analysis. Oral Surg. Oral Med. Oral Pathol. Oral Radiol..

[29] Brown J.S., Lowe D., Kanatas A., Schache A. (2017). Mandibular reconstruction with vascularised bone flaps: A systematic review over 25 years. Br. J. Oral Maxillofac. Surg..

[30] Okuyama K., Michi Y., Mizutani M., Yamashiro M., Kaida A., Harada K. (2016). Clinical study on mandibular fracture after marginal resection of the mandible. Oral Surg. Oral Med. Oral Pathol. Oral Radiol..

[31] Vallittu P.K., Närhi T.O., Hupa L. (2015). Fiber glass-bioactive glass composite for bone replacing and bone anchoring implants. Dent. Mater..

[32] Seto I., Marukawa E., Asahina I. (2006). Mandibular reconstruction using a combination graft of rhBMP-2 with bone marrow cells expanded in vitro. Plast. Reconstr. Surg..

[33] Herford A.S., Boyne P.J. (2008). Reconstruction of mandibular continuity defects with bone morphogenetic protein-2 (rhBMP-2). J. Oral Maxillofac. Surg..

[34] Lommen J., Schorn L., Landers A., Holtmann H., Berr K., Kübler N.R., Sproll C., Rana M., Depprich R. (2019). Release kinetics of the model protein FITC-BSA from different polymer-coated bovine bone substitutes. Head Face Med..

